# Transient effects of chemotherapy for testicular cancer on mouse behaviour

**DOI:** 10.1038/s41598-020-67081-8

**Published:** 2020-06-23

**Authors:** Veronika Borbélyová, Emese Renczés, Michal Chovanec, Michal Mego, Peter Celec

**Affiliations:** 1 Institute of Molecular Biomedicine, Faculty of Medicine, Comenius University, Bratislava, Slovakia; 2 2nd Department of Oncology, Comenius University, Faculty of Medicine & National Cancer Institute, Bratislava, Slovakia; 3 Translational Research Unit, 2nd Department of Oncology, Comenius University, Faculty of Medicine & National Cancer Institute, Bratislava, Slovakia; 4 Department of Medical Oncology, National Cancer Institute, Bratislava, Slovakia; 5 Institute of Pathophysiology, Faculty of Medicine, Comenius University, Bratislava, Slovakia; 6 Department of Molecular Biology, Faculty of Natural Sciences, Comenius University, Bratislava, Slovakia

**Keywords:** Oncology, Chemotherapy, Neuroscience

## Abstract

The treatment of testicular cancer includes unilateral orchiectomy and chemotherapy and is curative for most patients. However, observational studies revealed an association with depression, anxiety and cognitive impairment. It is unclear whether these side effects are caused by chemotherapy, hemicastration or the disease itself. The aim of our study was to analyse the behavioural effects of hemicastration and chemotherapy in adult male mice. The animals were randomly divided into four groups – control, chemotherapy, hemicastration and hemicastration with chemotherapy. After chemotherapy that included three cycles of bleomycin, etoposide, cisplatin mice underwent a battery of behavioural tests. To assess the long-term effects animals were tested also 3 months after the end of treatment. Chemotherapy led to lower locomotor- and exploratory activity, higher anxiety-like behaviour and worse spatial memory immediately after treatment. These behavioural effects were not present three months later. Hemicastration had no effect on most of the observed outcomes. In conclusion, adverse behavioural effects induced by chemotherapy in mice are transient and disappear later in life. Further studies are needed to elucidate the mechanisms responsible for the observed effects.

## Introduction

Testicular cancer primarily affects young men at reproductive age^1^. Nowadays, testicular cancer is highly curable due to advances in treatment for testicular cancer^2,3^. Primary surgery (orchiectomy) followed by three to four cycles of multiagent chemotherapy (bleomycin, etoposide and cisplatin) has been considered as the standard first-line treatment of metastatic testicular cancer^4^. Despite high efficacy of this type of testicular cancer treatment, its adverse effects on quality of life were also reported. Besides deleterious effect of chemotherapy on reproductive function of men^5,6^, adverse behavioural effects including higher levels of anxiety and depression^7,8^, fatigue and cognitive impairment^9–11^ were also indicated. Clinical studies suggest that the mentioned adverse effects of chemotherapy are long-lasting – higher anxiety and depression are present even 11 years following the chemotherapy treatment^12,13^. Amidi *et al*.^9^ have observed cognitive impairment (impaired working memory, visual learning and memory, executive functioning, verbal learning and memory, processing speed) in testicular cancer patients 2–7 years following treatment. Chovanec *et al*.^14^ have reported a decrease in overall cognitive function score (sum of four subscales: perceived cognitive impairment and cognitive abilities, quality of life affected by cognitive impairment, cognitive impairment perceived by others) in patients 5–32 years following chemotherapy and radiotherapy treatment^15^. On the contrary, some studies have brought different results^16,17^ indicating the need to study the effects in controlled experimental conditions. Animal studies have shown gonadotoxic effects of chemotherapy for testicular cancer on the morphology and function of male rat reproductive system^18,19^, however, animal studies dealing with effects of multiagent chemotherapy on behaviour are still lacking. In addition, the behavioural effect of partial or compensated hypogonadism in clinical or experimental studies with or without chemotherapy has not been studied yet.

Although multiagent chemotherapy is very effective, it is not clear, whether adverse behavioural effects are due to chemotherapy, orchiectomy or cancer itself. To the best of our knowledge, no experimental studies have analyzed the effect of therapeutically relevant doses of chemotherapy on complex behavioural phenotype of mice. In this view, the aim of the present study was to describe behavioural effects of hemicastration and multiagent chemotherapy used for the treatment of testicular cancer in mice. In addition, long-term effects of multiagent chemotherapy were observed.

## Results

### Behavioural phenotyping at the end of application of chemotherapy

#### Locomotor activity

The locomotor activity (Fig. 2) was found to significantly differ between the groups. Two-way ANOVA revealed a main effect of treatment (F_1,51_ = 17, p < 0.001) but not surgery approach (F_1,51_ = 0.002, p = 0.97). The interaction between these two factors was statistically not significant (F_1,51_ = 0.04, p = 0.85, Fig. 2). Bonferroni corrected post hoc test showed that application of chemotherapy leads to reduced distance moved in both, SHAM (t_51_ = 3.08, p < 0.05) and hGDX groups (t_51_ = 2.75, p < 0.05) compared to control animals.

**Figure 1:**
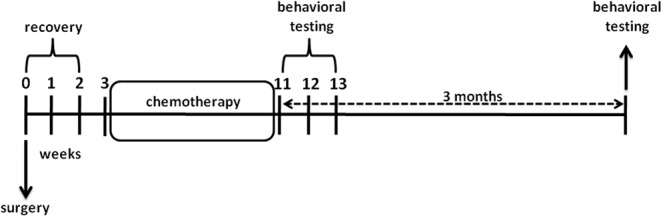
Timeline of the experiment. The schematic representation of the experimental design.

**Figure 2:**
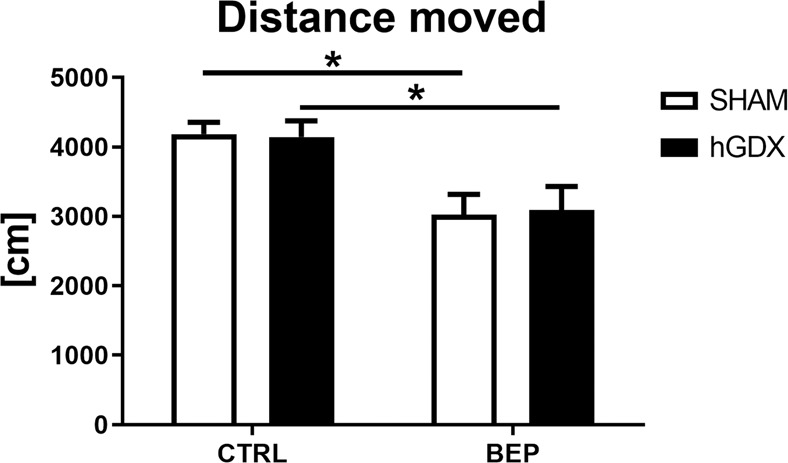
Locomotor activity in open field test. Application of chemotherapy led to lower locomotor activity as measured by total distance moved in both, SHAM and hGDX groups compared to CTRLs. Data are expressed as means + SEM. *p < 0.05. SHAM – sham operated mice, hGDX – mice with unilateral gonadectomy, CTRL – mice injected with saline, BEP – mice injected with three cycles of bleomycin, etoposide, and cisplatin. n = 15 mice for each group.

### Anxiety-like behaviour

**Figure 3:**
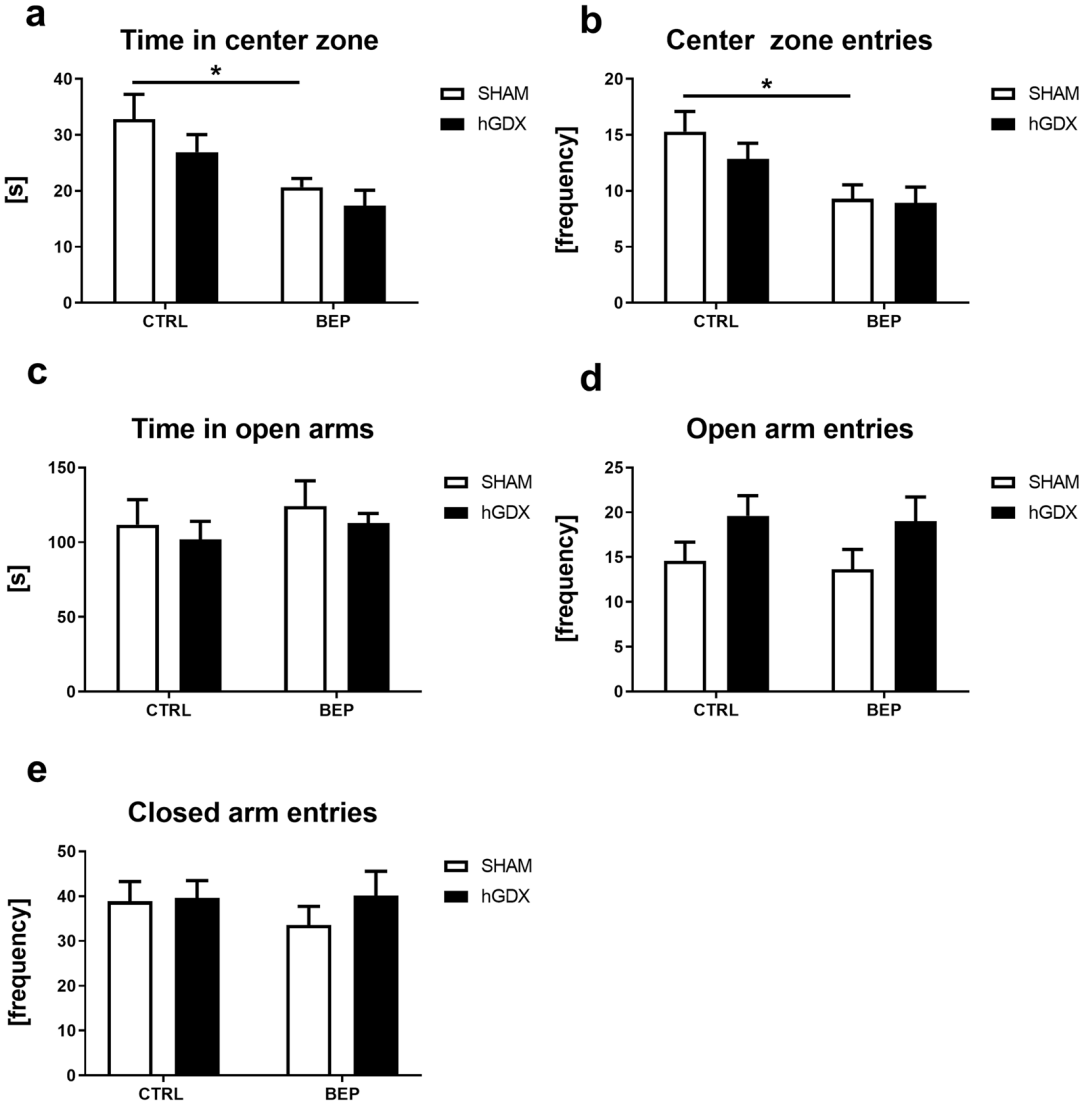
Anxiety-like behaviour in open field (**a,b**) and elevated plus maze test (**c,d**). General activity of mice in elevated plus maze test (**e**). Chemotherapy induced higher anxiety-like behaviour, an effect seen only in SHAM operated group of animals in open field test (**a,b**). No group differences were observed in any of parameters measured in elevated plus maze test (**c–e**). Data are expressed as means + SEM. *p < 0.05. SHAM – sham operated mice, hGDX – mice with unilateral gonadectomy, CTRL – mice injected with saline, BEP – mice injected with three cycles of bleomycin, etoposide, and cisplatin. n = 15 mice for each group.

Anxiety-like behaviour was observed in open field and elevated plus maze test. In open field test, two-way ANOVA indicated a main effect of treatment (F_1,51_ = 12.0, p < 0.01) but not surgery approach (F_1,51_ = 2.13, p = 0.15) on time spent in centre zone of the open field arena. The interaction between these two factors was statistically not significant (F_1,51_ = 0.18, p = 0.68, Fig. 3a). Bonferroni corrected post hoc test showed that BEP mice spent less time in centre zone than CTRL mice (t_51_ = 2.78, p < 0.05, Fig. 3a), an effect seen only in SHAM operated group (Fig. 3a). Similarly, a main effect of treatment (F_1,51_ = 11.1, p < 0.01) without a significant effect of surgery approach (F_1,51_ = 0.88, p = 0.35) was revealed using two-way ANOVA on number of entries into centre zone. The interaction between these two factors was statistically not significant (F_1,51_ = 0.49, p = 0.49, Fig. 3b). Bonferroni corrected post hoc test indicated that BEP mice entered the centre zone of the open field less frequently compared to CTRL mice (t_51_ = 2.88, p < 0.05, Fig. 3b).

In elevated plus maze test, there was no statistically significant effect observed when looking at treatment, surgery approach or their interaction on time spent in open arms [treatment: (F_1,51_ = 0.72, p = 0.40), surgery approach: (F_1,51_ = 0.58, p = 0.45), interaction: (F_1,51_ = 0.002, p = 0.96), Fig. 3c]. A main effect of surgery approach (F_1,51_ = 4.94, p < 0.05) without a significant effect of treatment (F_1,51_ = 0.11, p = 0.74) was observed in the number of open arm entries. The interaction between surgery approach and treatment was not significant (F_1,51_ = 0.04, p = 0.95, Fig. 3d). Following the Bonferroni post-hoc correction, no significant differences were observed between groups (Fig. 3c,d). There were no significant differences in general activity of mice measured as number of closed arm entries in the elevated plus maze test (effect of treatment: F_1,51_ = 0.28, p = 0.60, surgery approach: F_1,51_ = 0.65, p = 0.42, treatment x surgery interaction: F_1,51_ = 0.41, p = 0.52, (Fig. 3e).

### Exploratory activity and memory

**Figure 4:**
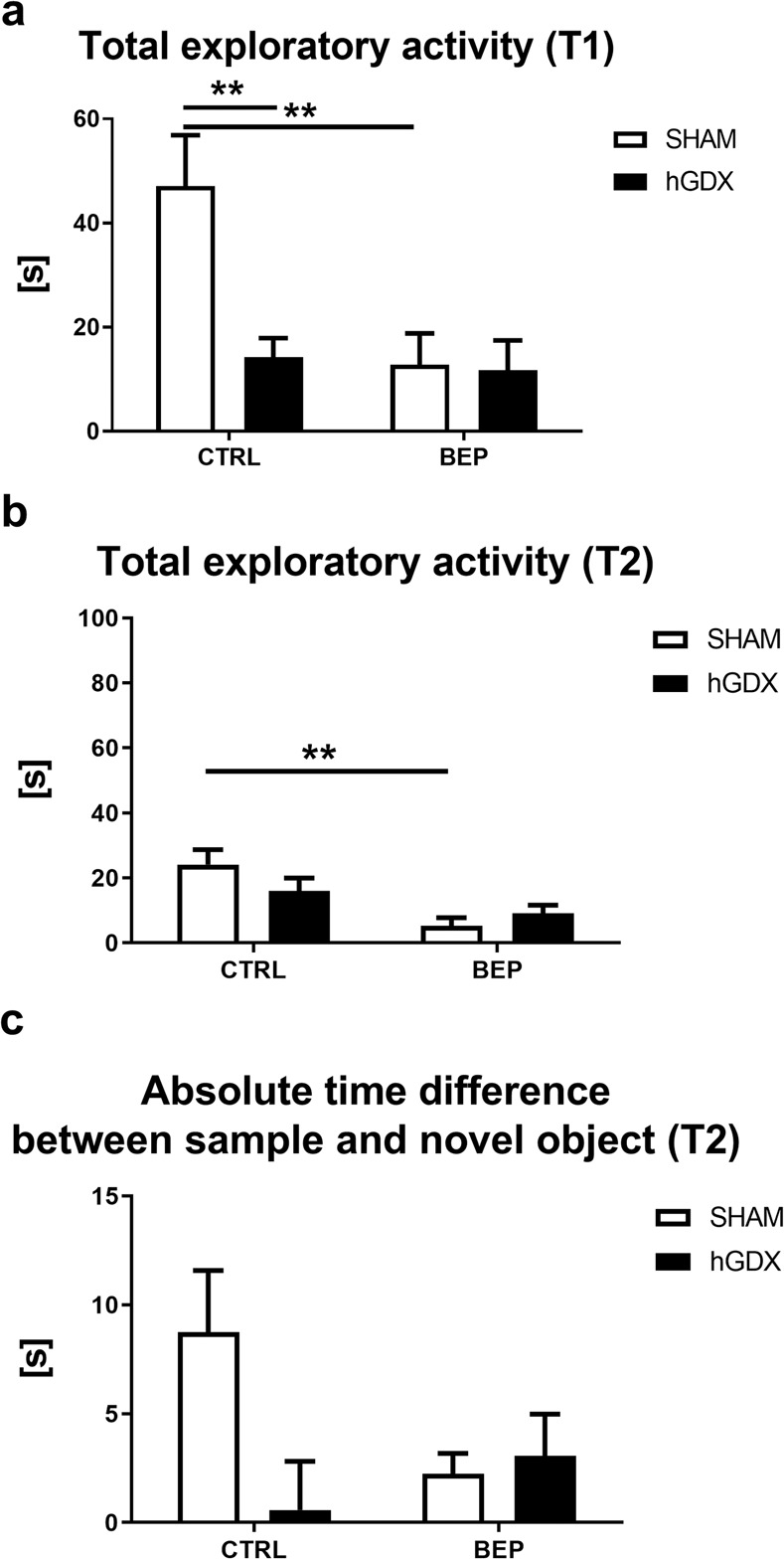
Exploratory activity (**a,b**) and memory (**c**) in the novel object recognition test. Chemotherapy led to lower exploratory activity during both trials of the novel object recognition test. This effect of chemotherapy was detected only in the SHAM group (**a,b**). Hemicastration had suppressive effect on total exploratory activity only in the first trial of the test (**a**). No group differences were observed in absolute time difference between interacting the sample and novel object (**c**). Data are expressed as means + SEM. **p < 0.01. SHAM – sham operated mice, hGDX – mice with unilateral gonadectomy, CTRL – mice injected with saline, BEP – mice injected with three cycles of bleomycin, etoposide, and cisplatin, T1 and T2 – the first and second trial of the novel object recognition test. n = 15 mice for each group.

In the first trial of the novel object recognition test, a main effect of treatment (F_1,51_ = 7.45, p < 0.01) and surgery approach (F_1,51_ = 6.37, p < 0.05) on total exploratory activity of mice was observed. The interaction between these two factors was significant (F_1,51_ = 5.58, p < 0.05, Fig. 4a). Bonferroni post-hoc correction showed that BEP mice spent less time interacting with objects compared to CTRL mice (t_51_ = 3.64, p < 0.01, Fig. 4a). In the first trial of the test, hGDX mice spent less time with exploring objects than CTRL mice (t_51_ = 3.42, p < 0.01, Fig. 4a).

Similarly, in the second trial, a significant effect of treatment (F_1,51_ = 13.4, p < 0.001) but not surgery approach (F_1,51_ = 0.39, p = 0.54) on total exploratory activity was observed. The interaction between these factors (F_1,51_ = 2.93, p = 0.09, Fig. 4b) was not statistically significant. Following the Bonferroni post-hoc correction, BEP mice spent less time interacting with objects compared to CTRL mice (t_51_ = 3.83, p < 0.01, Fig. 4b).

In addition, the absolute time difference between investigating the sample and novel object was calculated in the second trial. There was no statistically significant effect observed when looking at treatment (F_1,51_ = 0.92, p = 0.34) and surgery approach (F_1,51_ = 3.07, p = 0.09). The interaction between these factors was significant (F_1,51_ = 4.61, p < 0.05). Following the Bonferroni post-hoc correction, no significant differences were observed between groups (Fig. 4c).

### Spatial recognition memory

**Figure 5:**
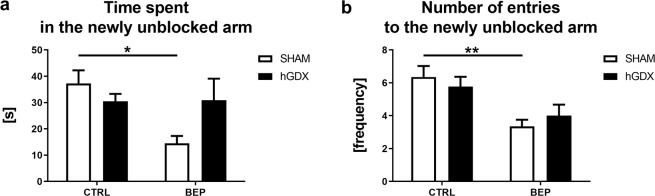
Spatial recognition memory in the T-maze. Chemotherapy led to impaired spatial recognition memory. This effect of chemotherapy was observed only in SHAM group of animals (**a,b**). Data are expressed as means + SEM. *p < 0.05 and **p < 0.01. SHAM – sham operated mice, hGDX – mice with unilateral gonadectomy, CTRL – mice injected with saline, BEP – mice injected with three cycles of bleomycin, etoposide, and cisplatin. n = 15 mice for each group.

In the second trial of the T maze test, a main effect of treatment (F_1,51_ = 4.54, p < 0.05) but not surgery approach (F_1,51_ = 0.84, p = 0.37) on time exploring the newly unblocked arm was detected. The interaction between these two factors was significant (F_1,51_ = 4.84, p < 0.05, Fig. 5a). Bonferroni corrected post hoc test showed that BEP mice spent less time exploring the newly unblocked arm compared to CTRL mice (t_51_ = 3.09, p < 0.05, Fig. 5a), an effect observed only in SHAM group of animals.

Similarly, a main effect of treatment (F_1,51_ = 15.9, p < 0.001) without a significant effect of surgery approach (F_1,51_ = 0.002, p = 0.96) was revealed using two-way ANOVA on number of entries into previously blocked arm. The interaction between these two factors was statistically not significant (F_1,51_ = 1.06, p = 0.31, Fig. 5b). Bonferroni corrected post hoc test indicated that BEP mice entered the previously blocked arm less frequently compared to CTRL mice (t_51_ = 3.58, p < 0.01, Fig. 5b).

### Depression-like behaviour

**Figure 6:**
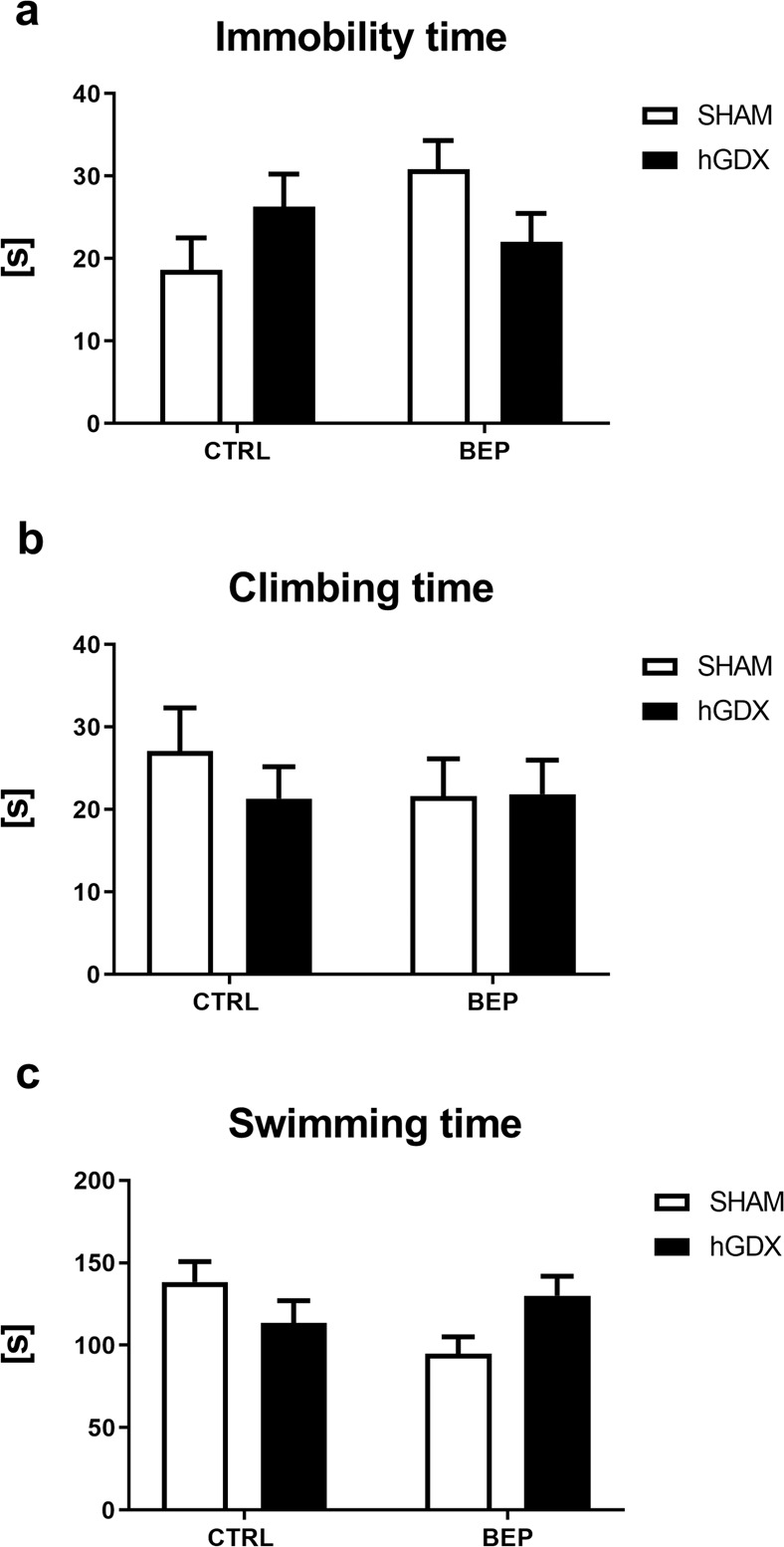
Depression-like behaviour, swimming and climbing behavior of mice in the forced swim test. Chemotherapy did not affect depression-like behaviour (**a**), climbing (**b**) and swimming **(c**) of mice. Data are expressed as means + SEM. SHAM – sham operated mice, hGDX – mice with unilateral gonadectomy, CTRL – mice injected with saline, BEP – mice injected with three cycles of bleomycin, etoposide, and cisplatin. n = 15 mice for each group.

No significant difference between the treatment groups was found in time spent immobile using two-way ANOVA [treatment (F_1,51_ = 1.15, p = 0.29), surgery approach (F_1,51_ = 0.03, p = 0.87), interaction (F_1,51_ = 4.91, p = 0.05), Fig. 6a] and time spent with climbing [treatment (F_1,51_ = 0.30, p = 0.59), surgery approach (F_1,51_ = 0.38, p = 0.54), interaction (F_1,51_ = 0.44, p = 0.51), Fig. 6b]. Regarding time spent with swimming (Fig. 6c), two-way ANOVA revealed a significant treatment x surgery approach interaction (F_1,51_ = 6.27, p < 0.05), while no main effect of either treatment (F_1,51_ = 1.27, p = 0.27) or surgery approach (F_1,51_ = 0.19, p = 0.67) on this type of behaviour was observed.

### Late onset effect of multiagent chemotherapy

#### Locomotor activity

**Figure 7:**
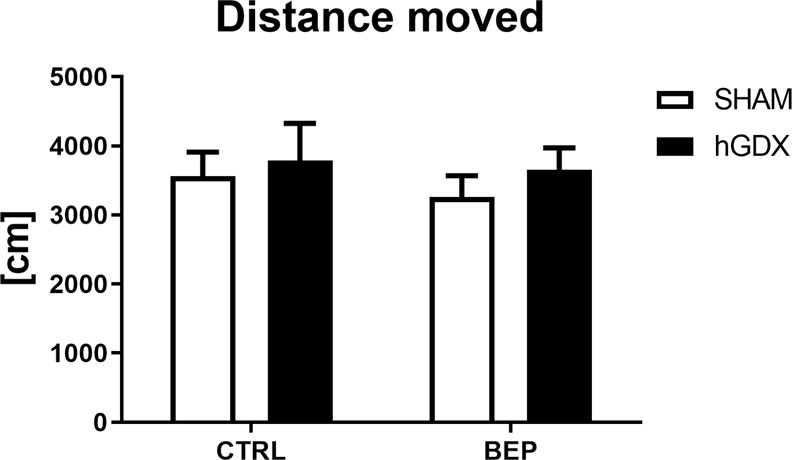
Late onset effect of chemotherapy on locomotor activity in open field test. The groups did not differ significantly in total distance moved in the open field arena. Data are expressed as means + SEM. SHAM – sham operated mice, hGDX – mice with unilateral gonadectomy, CTRL – mice injected with saline, BEP – mice injected with three cycles of bleomycin, etoposide, and cisplatin. n = 15 mice for each group.

In the open field test, there were no significant differences in the parameter of distance moved [effect of treatment (F_1,38_ = 0.35, p = 0.56), surgery approach (F_1,38_ = 0.71, p = 0.41), interaction (F_1,38_ = 0.05, p = 0.82, Fig. 7)] among groups.

### Anxiety-like behaviour

**Figure 8:**
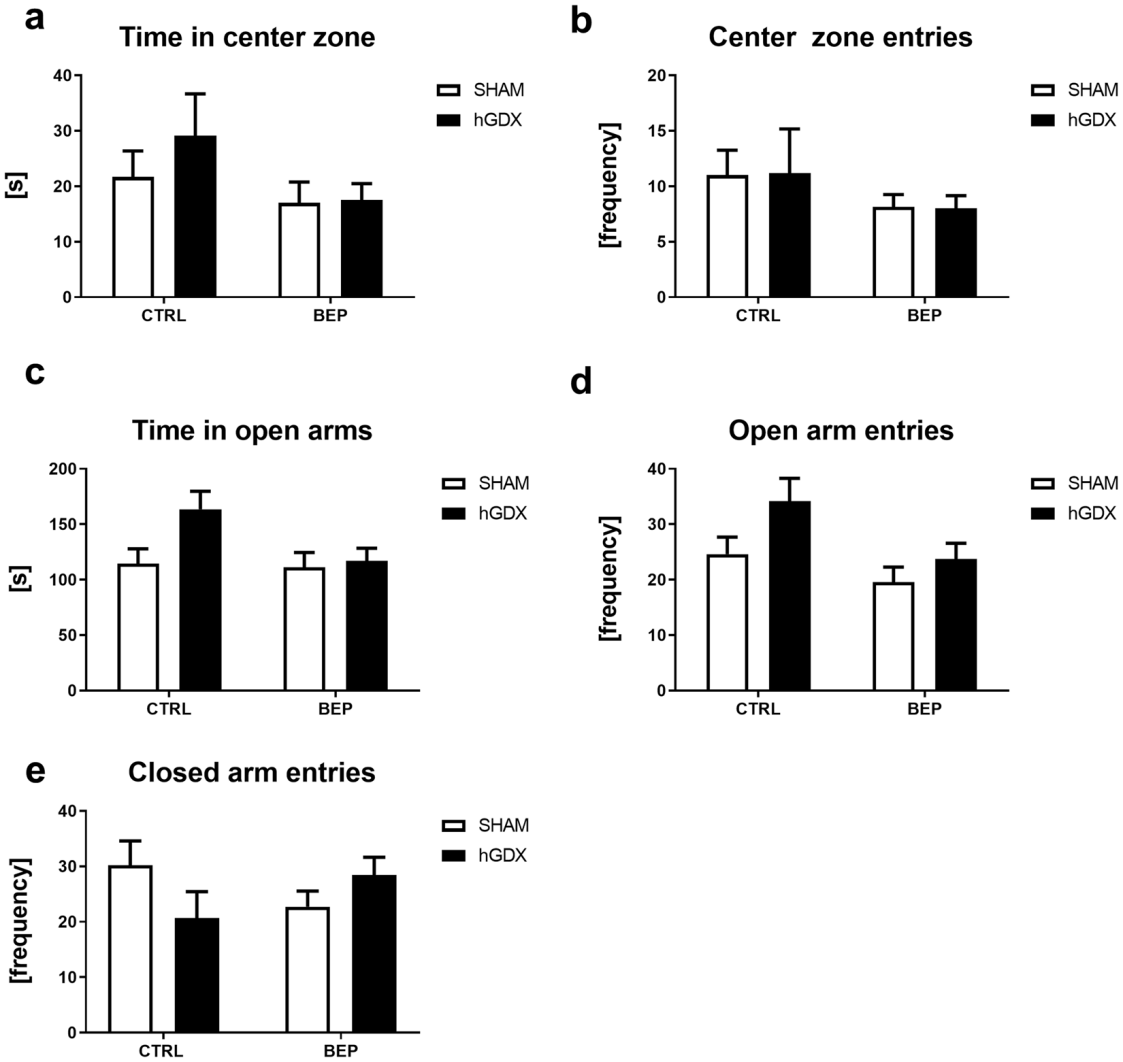
Late onset effect of chemotherapy on anxiety-like behaviour of mice in both, the open field (**a,b**) and elevated plus maze test (**c,d**). General activity of mice in elevated plus maze test (**e**). There were no significant differences in any of the observed behavioural parameters between groups. Data are expressed as means + SEM. SHAM – sham operated mice, hGDX – mice with unilateral gonadectomy, CTRL – mice injected with saline, BEP – mice injected with three cycles of bleomycin, etoposide, and cisplatin. n = 15 mice for each group.

There was no statistically significant effect observed when looking at treatment (F_1,38_ = 3.21, p = 0.08) and surgery approach (F_1,38_ = 0.78, p = 0.38) on time spent in centre of the open field arena (Fig. 8a). The interaction between these factors was not significant (F_1,38_ = 0.58, p = 0.45, Fig. 8a). Similarly, no group differences were observed in number of centre zone entries [treatment (F_1,38_ = 2.45, p = 0.13), surgery approach (F_1,38_ = 3.82, p = 0.99), interaction (F_1,38_ = 0.006, p = 0.94, Fig. 8b].

Regarding elevated plus maze test, there were no significant differences between groups of mice in time spent in open arms [treatment: (F_1,38_ = 3.05, p = 0.09), surgery approach: (F_1,38_ = 3.67, p = 0.06), interaction: (F_1,38_ = 2.30, p = 0.14), Fig. 8c]. A main effect of treatment (F_1,38_ = 5.73, p < 0.05) and surgery approach (F_1,38_ = 4.54, p < 0.05) on number of open arm entries was revealed by two-way ANOVA. The interaction between these two factors was not significant (F_1,38_ = 0.70, p = 0.41). Following the Bonferroni post-hoc correction, no significant differences were observed between groups (Fig. 8d). There were no significant differences in general activity of mice measured as number of closed arm entries (effect of treatment: F_1,38_ = 0.001, p = 0.97, surgery approach: F_1,38_ = 0.25, p = 0.62, treatment x surgery interaction: F_1,38_ = 3,98, p = 0.06, Fig. 8e).

### Exploratory activity and memory

**Figure 9:**
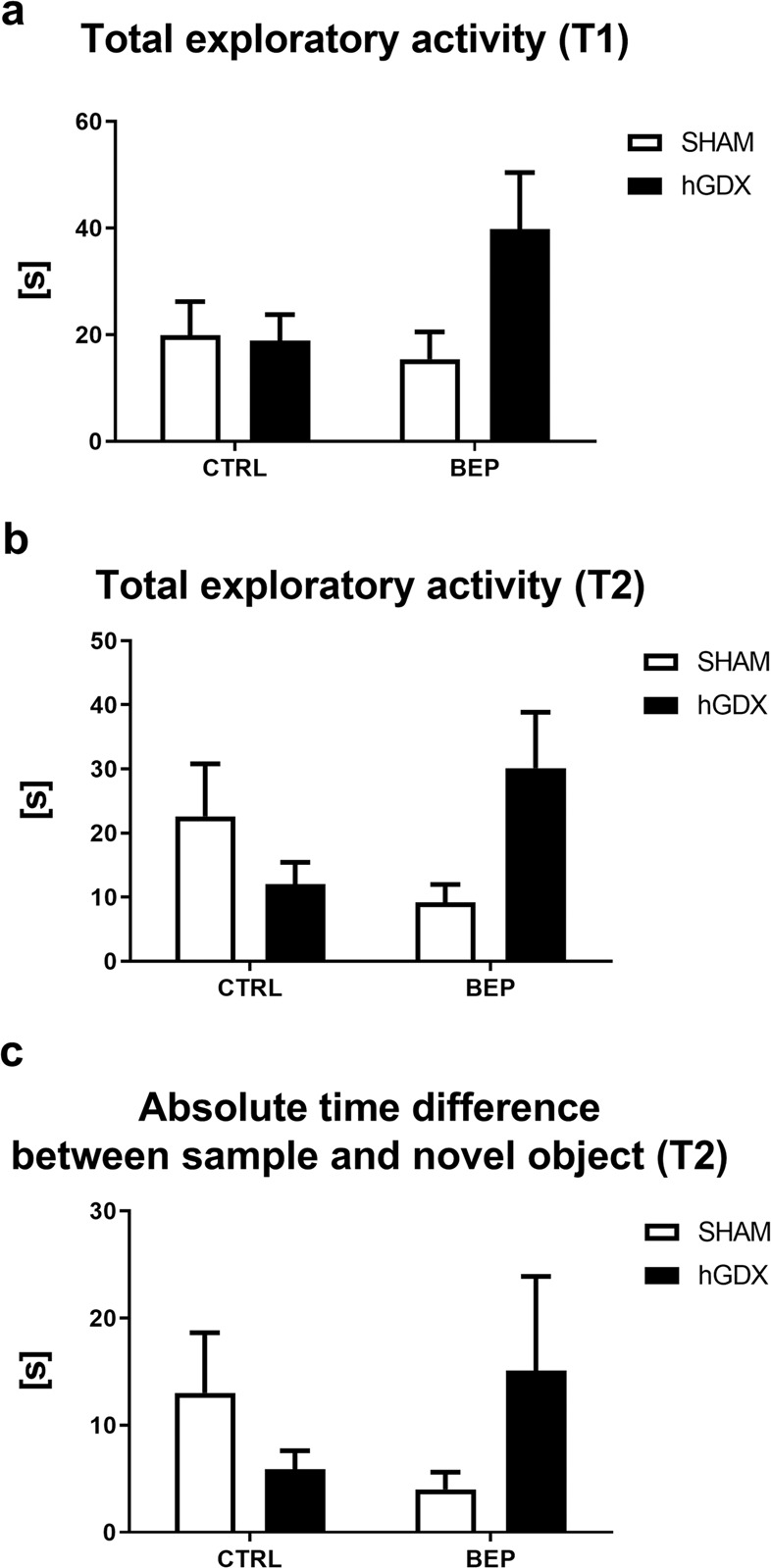
Late onset effect of chemotherapy on overall exploratory activity (**a,b**) and memory (**c**) in the novel object recognition test. No group differences were revealed in any of observed behavioural parameters. Data are expressed as means + SEM. SHAM – sham operated mice, hGDX – mice with unilateral gonadectomy, CTRL – mice injected with saline, BEP – mice injected with three cycles of bleomycin, etoposide, and cisplatin, T1 and T2 – the first and second trial of the novel object recognition test. n = 15 mice for each group.

No differences were found in the overall exploratory activity of mice in both, the first trial (Fig. 9a) and second trial (Fig. 9b) of the novel object recognition test. Regarding first trial, neither effect of treatment (F_1,38_ = 1.01, p = 0.32) nor surgery approach (F_1,38_ = 2.07, p = 0.16) was observed. The interaction between these two factors was not significant (F_1,38_ = 2.42, p = 0.13, Fig. 9a). During second trial, neither effect of treatment (F_1,38_ = 0.12, p = 0.74) nor surgery approach (F_1,38_ = 0.55, p = 0.46) was detected. The interaction between these two factors was not significant (F_1,38_ = 5.06, p = 0.05, Fig. 9b).

There were no group differences in absolute time difference between investigating the sample and novel object in the second trial. There was no statistically significant effect of treatment (F_1,38_ = 0.02, p = 0.99) or surgery (F_1,38_ = 0.10, p = 0.75). The interaction was not significant either (F_1,38_ = 2.20, p = 0.15, Fig. 9c).

### Spatial recognition memory

**Figure 10:**
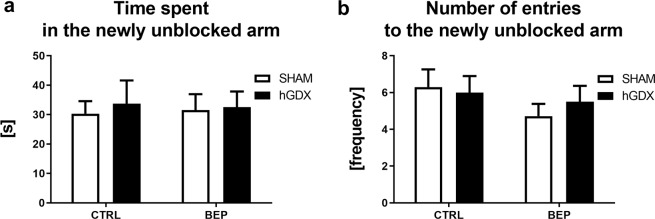
Late onset effect of chemotherapy on spatial recognition memory of mice in the T-maze assessed as time spent in the newly unblocked arm (**A**) or number of entries to the newly blocked arm (**B**). Chemotherapy did not affect spatial recognition memory of mice. Data are expressed as means + SEM. SHAM – sham operated mice, hGDX – mice with unilateral gonadectomy, CTRL – mice injected with saline, BEP – mice injected with three cycles of bleomycin, etoposide, and cisplatin. n = 15 mice for each group.

There were no differences in spatial recognition memory of mice 3 months after the end of chemotherapy. Neither an effect of treatment (F_1,38_ = 5.06, p = 0.99) nor surgery (F_1,38_ = 0.14, p = 0.71) on time spent with exploring the newly unblocked arm was observed. The interaction between these two factors was not significant (F_1,38_ = 0.04, p = 0.84, Fig. 10a). Similarly, there were no differences in number of entries to the previously blocked arm among groups. Two-way ANOVA indicated no significant effect of treatment (F_1,38_ = 1.37, p = 0.25) or surgery approach (F_1,38_ = 0.07, p = 0.79) on this behavioural parameter. The interaction between these two factors was statistically not significant (F_1,38_ = 0.37, p = 0.55, Fig. 10b).

### Depression-like behaviour

**Figure 11:**
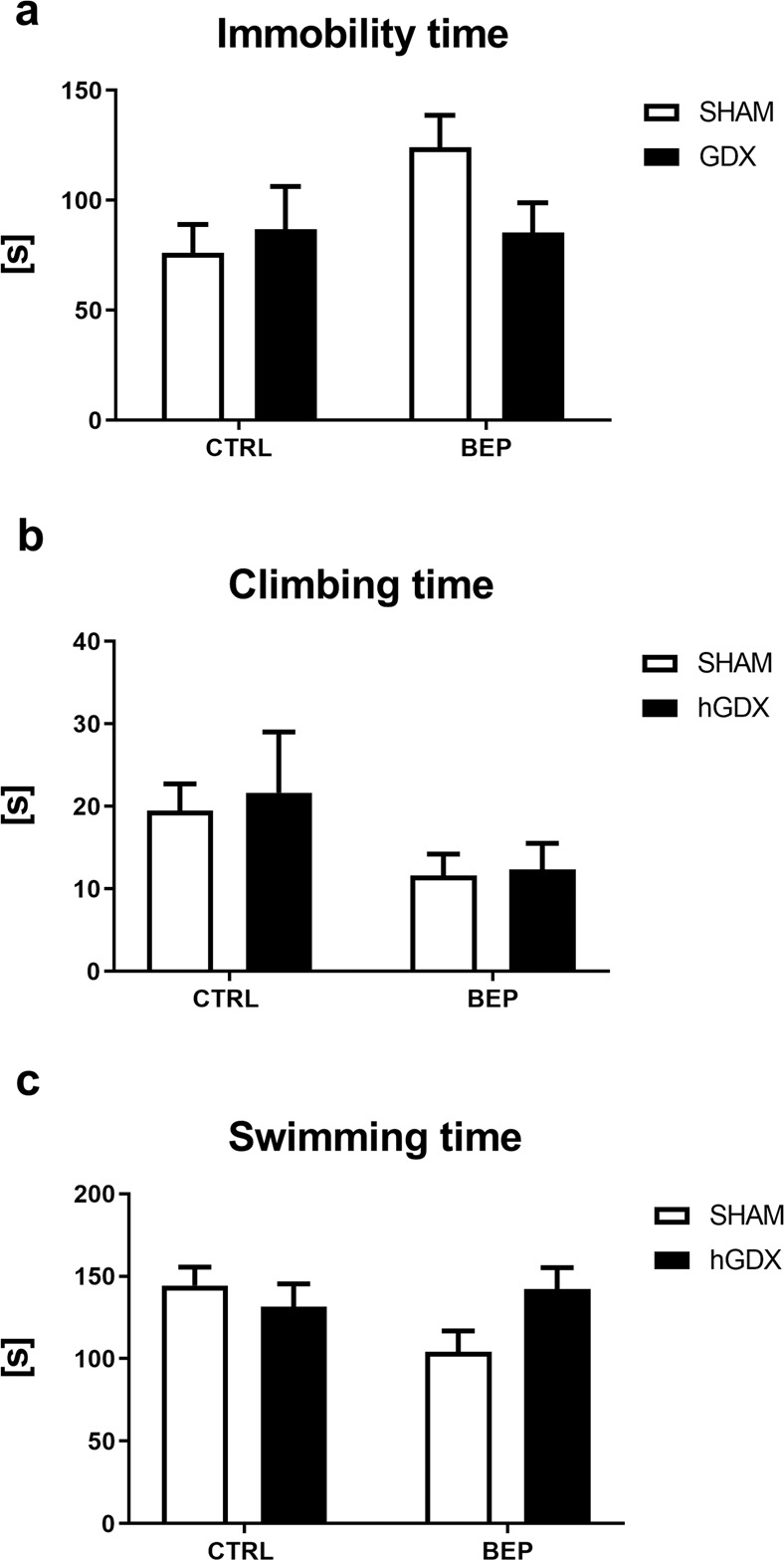
Late onset effect of chemotherapy on depression-like behaviour, swimming and climbing behavior of mice in the forced swim test. Chemotherapy did not affect depression-like behaviour (**a**), climbing (**b**) and swimming (**c**) of mice 3 months after the end of the treatment. Data are expressed as means + SEM. SHAM – sham operated mice, hGDX – mice with unilateral gonadectomy, CTRL – mice injected with saline, BEP – mice injected with three cycles of bleomycin, etoposide, and cisplatin. n = 15 mice for each group.

Chemotherapy had no effect on depression-like behaviour of mice 3 months after the end of chemotherapy application. Two-way ANOVA indicated neither an effect of treatment (F_1,38_ = 2.22, p = 0.14) nor surgery (F_1,38_ = 0.81, p = 0.37) on time spent immobile. The interaction between these factors was not significant (F_1,38_ = 2.50, p = 0.12, Fig. 11a). No group differences were observed in time spent swimming during the forced swim test [effect of treatment (F_1,38_ = 1.14, p = 0.29), surgery approach (F_1,38_ = 0.84, p = 0.37), and treatment x surgery interaction (F_1,38_ = 3.42, p = 0.07, Fig. 11c]. Climbing activity of mice was affected by chemotherapy (F_1,38_ = 5.23, p < 0.05), but not by surgery approach (F_1,38_ = 0.15, p = 0.70). The treatment x surgery approach interaction was not significant (F_1,38_ = 0.04, p = 0.85, Fig. 11b). No significant differences were observed in any of the observed behavioural parameters between the groups.

### Working memory and reference memory

**Figure 12:**
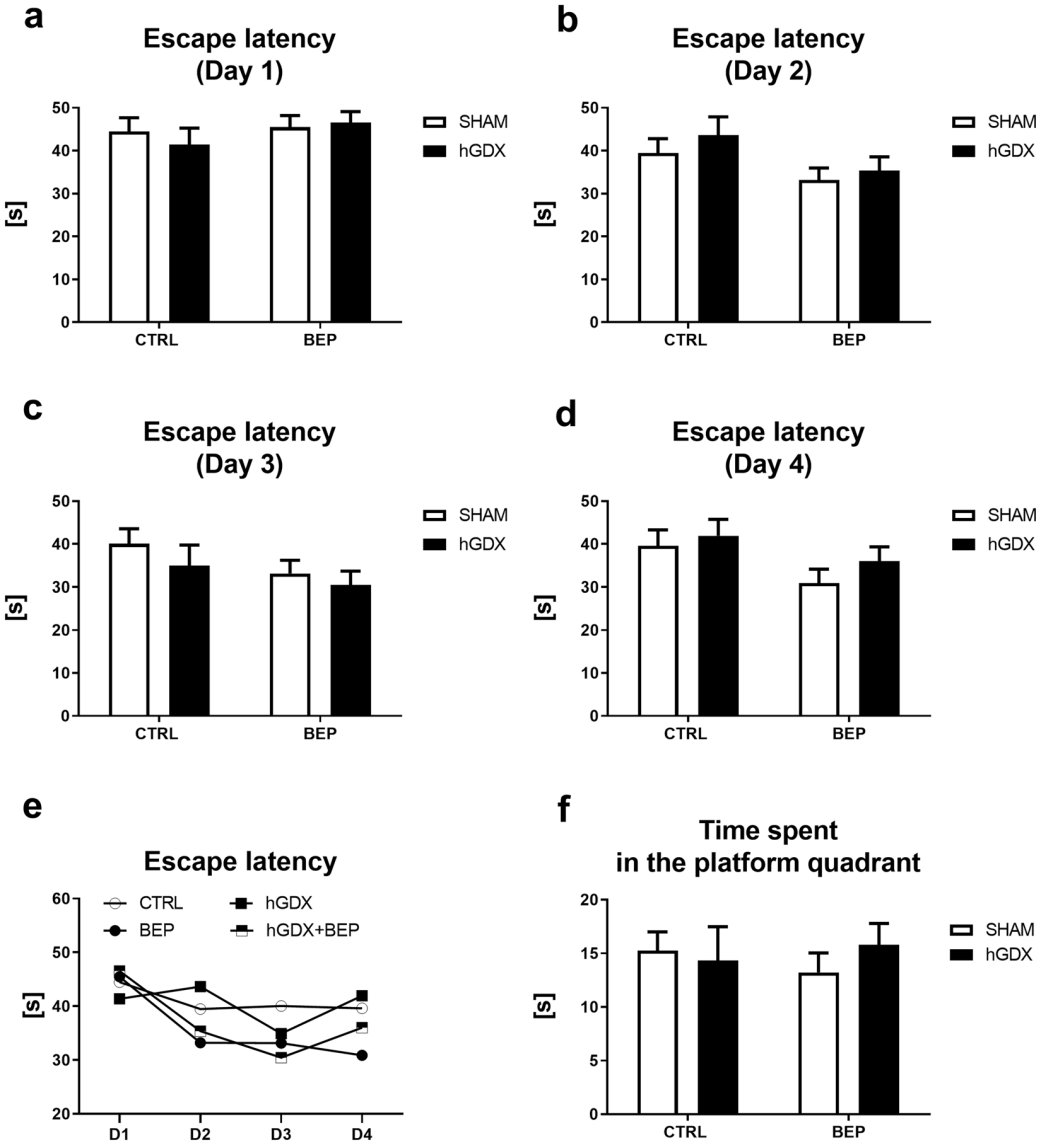
Late onset effect of chemotherapy on working (**a–e**) and reference (**f**) memory of mice in Morris water maze task. Chemotherapy did not affect working (**a–e**) and reference memory of mice (**f**). No differences were found between groups in daily escape latencies (**a–d**). Animals showed general improvement from day 1 to day 4 of the testing (**e**). Data are expressed as means + SEM. SHAM – sham operated mice, hGDX – mice with unilateral gonadectomy, CTRL – mice injected with saline, BEP – mice injected with three cycles of bleomycin, etoposide, and cisplatin, D1, D2, D3, D4 – the first, second, third and fourth day of testing. n = 15 mice for each group.

In the Morris water maze test, there were no group differences in short-term working memory of mice. All groups performed similarly during the individual days of the acquisition part of the test (Fig. 12a–e). Escape latencies calculated for day 1 (Fig. 12a) and 3 (Fig. 12c) of acquisition period did not show a significant effect of treatment and surgery [day 1: treatment (F_1,17_ = 1.00, p = 0.32), surgery (F_1,17_ = 1.00, p = 0.32); day 3: treatment (F_1,17_ = 2.45, p = 0.12), surgery (F_1,17_ = 1.16, p = 0.28)]. There was no significant treatment x surgery interaction for escape latencies on these days of testing [day 1: interaction (F_1,17_ = 0.46, p = 0.50); day 3: interaction (F_1,17_ = 0.11, p = 0.74)].

A main effect of treatment [day 2: (F_1,17_ = 4.63, p < 0.05); day 4: (F_1,17_ = 4.03, p < 0.05)] without a significant effect of surgery [day 2: (F_1,17_ = 0.88, p = 0.35); day 4: (F_1,17_ = 1.05, p = 0.31)] was observed in the escape latency time on the 2^nd^ (Fig. 12b) and 4^th^ day (Fig. 12d) of spatial ability testing. The interaction between treatment and surgery approach was also not significant [day 2: (F_1,17_ = 0.09, p = 0.77); day 4: (F_1,17_ = 0.15, p = 0.70)]. Bonferroni post-hoc correction did not show any group differences.

Regarding long-term reference memory testing (probe trial, Fig. 12f), all groups spent equal time in the platform quadrant [(mean ± SEM: CTRL (15.3 ± 1.76); BEP: (13.2 ± 1.81); hGDX: (14.3 ± 3.16); hGDX+BEP: (15.8 ± 1.99)]. No effect of treatment (F_1,38_ = 0.02, p = 0.90) and surgery approach (F_1,38_ = 0.15, p = 0.70) was observed. The treatment x surgery approach interaction was not significant (F_1,38_ = 0.65, p = 0.42). Bonferroni post-hoc correction did not show any group differences.

**Figure 13:**
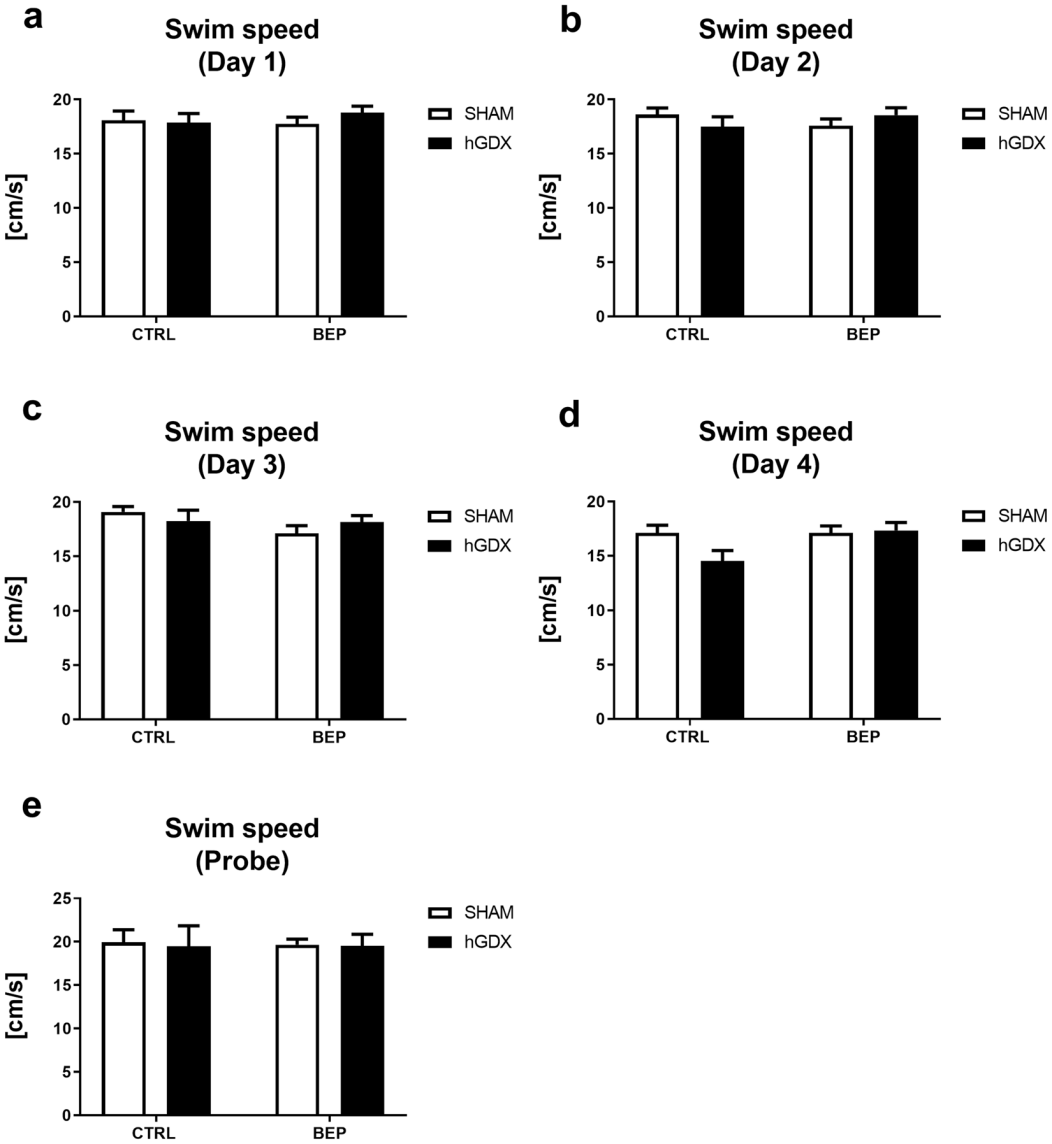
Late onset effect of chemotherapy on swim speed (**a–e**) of mice in Morris water maze task. Chemotherapy did not affect swim speed of mice. No differences were found between groups in daily measured swim speed. Data are expressed as means + SEM. SHAM – sham operated mice, hGDX – mice with unilateral gonadectomy, CTRL – mice injected with saline, BEP – mice injected with three cycles of bleomycin, etoposide, and cisplatin. n = 15 mice for each group.

No differences between the groups were found in the ability to swim (Fig. 13). The ability of mice to swim calculated as mean swim velocity for day 1 (Fig. 13a), day 2 (Fig. 13b), day 3 (Fig. 13c), day 4 (Fig. 13d) and probe trial (Fig. 13e) did not show a main effect of treatment [day 1: F_1,168_ = 0.15, p = 0.69; day 2: F_1,168_ = 9,9 × 10^−5^, p = 0.99; day 3: F_1,168_ = 2.08, p = 0.15; day 4: F_1,168_ = 3.44, p = 0.07; probe: F_1,38_ = 0.008, p = 0.93] and surgery [day 1: F_1,168_ = 0.31, p = 0.58; day 2: F_1,168_ = 0.009, p = 0.92; day 3: F_1,168_ = 0.01, p = 0.91; day 4: F_1,168_ = 2.39, p = 0.12; probe: F_1,38_ = 0.04, p = 0.84]. The treatment x surgery interaction was not significant [day 1: F_1,168_ = 0.76, p = 0.38; day 2: F_1,168_ = 2.11, p = 0.15; day 3: F_1,168_ = 1.64, p = 0.20; day 4: F_1,168_ = 3.31, p = 0.07; probe: F_1,38_ = 0.01, p = 0.92].

## Discussion

The link between testicular cancer treatment and decline in quality of life including anxiety, depression, fatigue and cognitive difficulties is evident from clinical observational studies in testicular cancer survivors^7–10,14^. However, it is unclear whether these adverse behavioural effects are caused by chemotherapy, hemicastration or by the treated cancer. This study aimed to evaluate the effect of hemicastration and multiagent chemotherapy used for the treatment of testicular cancer in adult male mice. Administration of bleomycin, etoposide, cisplatin chemotherapy led to lower locomotor- and exploratory activity, higher anxiety-like behaviour and worse spatial memory in mice tested after the end of the third cycle of the treatment. However, chemotherapy did not affect depression-like behaviour of mice and more importantly, the observed adverse behavioural effects were not detected three months later. Experimental groups of mice displayed similar general activity in the elevated plus maze test and also equal swim speed in the Morris water maze test, suggesting that the observed behavioural effects are indeed direct effects of chemotherapy. Thus, anxiety-like behaviour was not affected by motor impairment. On contrary, we can speculate that chemotherapy-induced increase of anxiety-like behaviour could affect the exploratory and locomotor activity^20,21^. Our results are in consensus with findings of previous animal studies reporting lower locomotor activity^22,23^, higher anxiety-like behaviour^24^ and impaired memory^24,25^ following chemotherapy. Chemotherapy-induced higher depression-like behaviour was reported by Iarkov *et al*.^25^, however, this effect of chemotherapy was not confirmed in our study. In comparison to our study, all of the aforementioned studies differ in methodological details including species and type of chemotherapeutics used, and also dosing, timing and regimen of chemotherapy exposure. Moreover, previously published studies e.g^22,24,25^. observed mainly the short-term effect of chemotherapy on animal behaviour, thus, our study is the first dealing with both, short- and long-term behavioural effects of multiagent chemotherapy. Hemicastration did not affect most of the observed outcomes.

Animal studies dealing with the behavioural effects of the most efficient multiagent chemotherapy in humans (co-administration of bleomycin, etoposide and cisplatin) are lacking. The only experimental study conducted on rats is the study of Kilarkaje *et al*.^19^ investigating the effect of multiagent chemotherapy on fertility and not on behaviour of the rats. To the best of our knowledge, our experiment is the first study investigating the effects of this type of chemotherapy on mouse behaviour. Animal models that appropriately mimic human testicular cancer treatment are extremely important. Furthermore, an experimental study allows investigation of the effect of chemotherapy and hemicastration on behaviour of mice separately, which is not possible in humans. Regarding the present study, several strengths should be pointed out. In our study, hemicastration of mice was performed to mimic surgical part of the treatment of testicular carcinoma in humans. Subsequently, the mice received multiagent chemotherapy according to the protocol widely used for treating human testicular cancer patients. In addition, to mimic clinical heterogenity present in clinical practice, outbred strain (CD-1) of mice was used. The animals underwent a comprehensive phenotyping using a battery of behavioural tests. However, there are several inherent limitations of this study. The animals were healthy, without any testicular tumours that might also affect observed behavioural outcomes. In 75% of testicular cancer patients after unilateral orchiectomy a so-called compensated hypogonadism with normal concentration of testosterone and elevated concentration of luteinizing hormone can be found^26^. A technical limitation of the present experiment is the lack of luteinizing hormone measurement after hemicastration, thus the confirmation of compensated hypogonadism in mice. In the present study, hemicastration and the likely induced compensated hypogonadism did not affect most of behavioural outcomes. Another limitation of our study may be a lack of use of antiemetic prophylaxis in experimental animals that is routinely used in testicular cancer patients. Such treatment may also modulate cognitive functions during and immediately after chemotherapy. In contrast to patients, in mice, the chemotherapeutic drugs are administered intraperitoneally. The route of administration could affect the pharmacokinetics and, thus, the results of the present study. It is, however, an integral limitation of all animal studies.

This study shows that chemotherapy affects the behaviour temporarily and does not cause any long-term behavioural disturbances in mice. Data from observational studies in testicular cancer survivors show worsened cognition immediately after chemotherapy and a subsequent return to normal after one year of follow up^27–29^. However, cognitive impairment was found again 7–10 years after chemotherapy^9,11,30^. Thus, we speculate that after a short-term normalization a long-term decrease of cognitive functioning would be found in mice as well. This must be proved in further longer studies. In addition, further research is needed to investigate the possible role of luteinizing hormone and the involved molecular mechanisms of the behavioural effects to identify possible therapeutic targets in testicular cancer survivors.

In conclusion, adverse behavioural effects induced by chemotherapy in mice were transient and disappeared later in life. Additional longer and more detailed studies are needed to elucidate the possible mechanisms in the long-term adverse behavioural effects of chemotherapy.

## Materials and Methods

### Animals and housing conditions

In the present study, CD1 mice (n = 60) were used. Young male mice (6-weeks old, Velaz, Prague, Czech Republic) were group-housed (3–5 per cage) throughout the study in polycarbonate cages (36.5 × 20.5 × 14 cm) and maintained in a temperature- (22 ± 2 °C) and humidity-controlled room (55 ± 10%) with 12:12 h light/dark schedule. Mice had access to standard food and water *ad libitum*. All experimental procedures were approved by the Ethical Committee of the Institute of Molecular Biomedicine, Comenius University, Bratislava and have been conducted in accordance with the EU Directive 2010/63/EU, and Slovak legislation.

### Experimental design

#### Surgery

Adult male mice were randomly divided into four groups: control (CTRL, n = 15), chemotherapy (BEP, n = 15), hemicastration (hGDX, n = 15) and hemicastration with chemotherapy (hGDX + BEP, n = 15). At 8 weeks of age, hGDX mice underwent unilateral gonadectomy under xylazine (intraperitoneal injection of 10 mg/kg, Xylariem inj, Riemser, Germany) and ketamine (intraperitoneal injection of 100 mg/kg, Narkamon inj, Bioveta, Czech Republic) anaesthesia. In hGDX mice, either right or left testis (cauda and caput epididymis) was extracted through a small incision made at the posterior tip of the scrotum. The vas deferens and spermatic blood vessels were ligated with a silk suture. Control mice were sham operated, where either right or left testis was gently removed from and then replaced into the scrotum. After surgery, mice were group-housed with their previous cage mates and had access to food and water *ad libitum*. The schematic representation of the experimental design is shown in Fig. 1.

### Application of multiagent chemotherapy

After a two-week recovery period, intraperitoneal application of chemotherapy was started. Experimental animals were treated with three cycles (21 days each) of bleomycin, etoposide and cisplatin (BEP) dissolved in saline according to the protocol of Kilarkaje *et al*.^19^. Briefly, bleomycin was injected on days 2, 9 and 16; while etoposide and cisplatin on days 1–5 of the given cycle. Control animals received saline.

### Behavioural testing after chemotherapy

At the end of the third cycle mice underwent a battery of behavioural tests assessing locomotor and exploratory activity, anxiety- and depression-like behaviour and memory. To evaluate potential late onset effects of chemotherapy, the animals underwent the same battery of behavioural tests 3 months after the end of treatment.

### Open field

The open field arena consisted of a square arena (45 cm × 45 cm). The arena was virtually divided into two zones, the central zone and border zone. Mice were placed into the centre of the arena and were allowed to freely explore it for 5 min. To evaluate locomotor activity, total distance moved was observed.

Parameters like the frequency of entrances and time spent in the centre zone were considered as anti-anxiety behaviour^31^.

### Novel object recognition

The novel object recognition task was conducted in the open field arena. The test had two sessions (5 min each) with retention interval of 1 hour. In this task, three different objects were used: 1. a standard plastic bottle, 2. a metallic can and 3. glass bottle. During the first session, the mice were placed in the middle of arena with two objects (plastic and metallic bottle) positioned at the opposite corners of the arena (27 cm from the wall and 55 cm apart of each other). Time spent exploring the object 1 or 2 was marked as “a1” or “a2”. During the second session, the plastic bottle was left in the arena (time “a3”) and the metallic can was replaced with a novel object (glass bottle, time “b”). Exploration was defined as sniffing or licking the objects. Total time exploring both objects during the first and second sessions (session 1: e1 = a1 + a2, session 2: e2 = a3 + b) and the absolute time difference between investigating the familiar and the novel object (session 2: d1 = b − a3) were recorded^32^.

### Elevated plus maze

The elevated plus maze apparatus consisted of four arms: two open without walls (45 cm × 10 cm) and two closed (45 cm × 10 cm) with 40 cm high walls. The four arms were extended from a central platform (10 cm × 10 cm). Each arm of the apparatus was attached to metal legs elevating the maze to a height of 50 cm above the floor. Mice were placed at the end of the open arm facing from the maze and were allowed to freely explore the maze for 5 min. The time spent in open arms and the number of open arm entries was considered to be indicators of anti-anxiety-like behaviour^33^. In addition, the number of closed arm entries was evaluated as an index of general activity^34^.

### T-maze

The T-maze apparatus consisted of three arms, including long arm (base: 10 cm × 40 cm × 20 cm) and two arms (10 cm × 30 cm × 20 cm) extending from the long arm of the maze. Spatial recognition memory was assessed using two-trial (5 min each) T-maze. The mice were started from the base of the T maze. In the first trial, one of the three arms was closed and mice had free access to explore two open arms during 5 min testing period. The retention interval was 30 min between the trials. During the second trial, all three arms of the maze were opened allowing the exploration of each arm for 5 min. To eliminate side preference, the position of the blocked arm was systematically altered between the animals. The observed parameter was the amount of time spent exploring and number of entries to the newly unblocked arm during the first two minutes of the second trial as an indicator of spatial recognition memory^35^.

### Forced swim test

Mice were individually placed into a transparent plastic cylinder (height 45 cm, diameter 30 cm) filled with tap water (23–25 °C). The time of immobility was recorded during 6 min testing period as an index of depression-like behaviour. Two additional parameters – time spent with swimming and climbing (escaping) were also evaluated. Due to the fact, that mice are very active at the beginning of the test, the first two minutes of the test was considered as habituation period and only the last four minutes of the test were analyzed^36^.

### Morris water maze

The Morris water maze consisted of a dark plastic circular pool (height 60 cm, diameter 125 cm) filled with water (25 ± 1 °C). The pool was virtually divided into four quadrants marked with intra-maze geometric cues (circle, square, triangle and star) inside the maze wall for orientation and spatial learning. A hidden circular platform (1 cm beneath the water surface) was placed in one of the four quadrants. The position of hidden platform (10 cm in diameter) remained unchanged during the 4-day acquisition period. The mice underwent four trials per day, always starting from a different quadrant. The order of the release positions was fixed during the acquisition phase of testing. One trial consisted of a swim, with up to 60 s duration, followed by a 30 s rest on the platform. If the mouse did not find the platform within 60 s, it was gently guided by an investigator to climb on platform. In acquisition period, escape latency (time to find the hidden platform) was calculated for each day to evaluate short-term working memory. On the 5^th^ day of testing, a probe trial was performed. The platform was removed and the mice were allowed to swim for 60 s. The release position was in the quadrant opposite to the platform-quadrant. The time spent in platform-quadrant was recorded as an indicator of long-term reference memory^37^. To assess whether mice in the groups are comparable in their ability to swim and in motivation to escape, mean swim velocity was calculated for each day of Morris water maze testing. In this study, we have focused on spatial learning ability (hippocampal function-dependent) of mice that is why we did not perform the visual platform learning trials assessing non-spatial learning ability (independent of hippocampal function) of animals. In addition, mice underwent Morris water maze testing only once, three months following the chemotherapy treatment to assess long-term effect of chemotherapy or castration without bias due to previous training/learning effects on spatial memory in the Morris water maze.

### Statistical analysis

Statistical analysis was performed using GraphPad Prism version 6.01 (GraphPad Software, Inc., CA, USA). To analyze the behaviour of mice, two-way analysis of variance (ANOVA, independent factors: treatment and surgery approach) with Bonferroni-corrected post-hoc t-test was used. P values lower than 0.05 were considered statistically significant. Data are presented as mean plus standard error of the mean (SEM).

## Data Availability

The datasets generated during the current study are available from the corresponding author on request.
